# Evolutionary Insight into Immunothrombosis as a Healing Mechanism

**DOI:** 10.3390/ijms23158346

**Published:** 2022-07-28

**Authors:** Eduardo Anitua, Roberto Prado, Sabino Padilla

**Affiliations:** 1 BTI—Biotechnology Institute I MAS D, 01007 Vitoria, Spain; eduardo@fundacioneduardoanitua.org (E.A.); roberto.prado@bti-implant.es (R.P.); 2 University Institute for Regenerative Medicine and Oral Implantology—UIRMI (UPV/EHU-Fundación Eduardo Anitua), 01007 Vitoria, Spain; 3 Eduardo Anitua Foundation for Biomedical Research, 01007 Vitoria, Spain

**Keywords:** evolution, healing, coagulation, hemostasis, platelets, fibrinogen, complement system, growth factors

## Abstract

Both invertebrates and vertebrates possess a cluster of immediate and local wound-sealing, pathogen-killing, and tissue healing responses known as immunoclotting and immunothrombosis, respectively, to cope with two life-threatening emergencies, namely, bleeding and microbial invasion. Despite their convergence in function, immunoclotting and immunothrombosis are deployed by different blood cells and intravascular multidomain proteins. In vertebrates, these proteins share some domains with intrinsic chemical affinities useful in generating cooperative networks such as pathogen and damage pattern recognition molecules. Moreover, many of the proteins involved in coagulation and fibrinolysis in humans are multifunctional molecules playing roles in other processes from inflammation to healing and beyond. In our modern society, however, the interaction of activated intravascular allosteric proteins with one another and with blood cells entails vulnerabilities posing a biological paradox: intravascular proteins that locally operate as tissue repair enhancers can nevertheless generate pathogenic processes by acting systemically. In this manuscript, we contextualize and frame the coagulation system and hemostasis through an evolutionary time scale, illustrating their role as dual players in the defense against exsanguination and pathogens while significantly influencing wound healing.

## 1. Introduction

Immunoclotting in invertebrates and immunothrombosis in vertebrates are responses evolutionarily linked to defense, clotting, and healing [1]. The main players involved in human immunothrombosis are the intravascular proteolytic cascade systems, including complement, coagulation, contact and fibrinolysis systems, and blood cells. The emergence and the domain organization of the proteins of intravascular proteolytic cascade systems show that these proteins were assembled by genetic variations of preexisting genes [2,3,4]. In addition to playing roles in coagulation and fibrinolysis, intravascular proteins of these two cascades and their catalytic and noncatalytic regions play multiple roles ranging from embryonic development and inflammation to healing and beyond [5]. However, in our modern society with cutting-edge therapeutic strategies, the interaction of activated intravascular proteins with one another and with blood cells entails vulnerabilities. This manuscript intends to frame the coagulation system and hemostasis through an evolutionary time scale, illustrating their dual roles in defense against exsanguination and pathogens while significantly influencing wound healing and the consequent tradeoffs in our modern society.

## 2. Convergence in Function of Invertebrate Immunoclotting and Vertebrate Immunothrombosis

**Figure 1:**
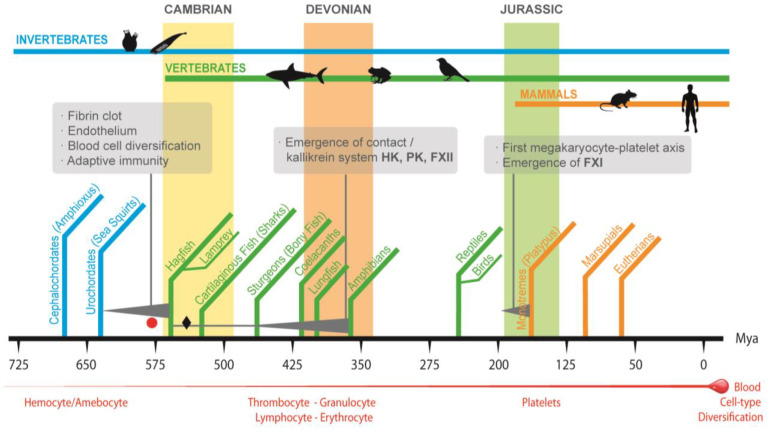
An overview of some of the main biological and environmental events in the evolution of the vertebrate coagulation system. The Cambrian radiation was preceded by a whole-genome duplication that, together with genetic variations in the form of gene duplication, exon shuffling, and simple mutation, led to a period of great innovations, including the emergence of organisms (the earliest jawless vertebrate agnathans similar to today’s hagfish and lampreys) with a simple version of coagulation system involving prothrombin, tissue factor, fibrinogen, FVII, and X. The emergence of the contact system and the integration into the vertebrate coagulation system was gradual, and it has been associated with the water-to-land transition of vertebrates and the appearance of the first amphibians somewhere around 400–390 Mya. The origin of the megakaryocyte/platelet axis (M/Pa) remains to be determined, but it might have occurred with the emergence of the first mammals around 200–166 Mya (the red circle and black diamond represent the points in evolution where the first and second whole-genome duplication events have been proposed to occur).

In invertebrates such as the American horseshoe crab, immunoclotting relies on soluble proteins with jellifying properties, including coagulogen, fondue, and hemolectin proteins, with scaffold-sealing and -healing functions [2,6]. In marine invertebrates such as sea urchins, however, coelomocytes aggregate upon injury or foreign substances such as lipopolysaccharide to form a cellular clot mediated by amassin, a plasma protein, whose multimers hook the coelomocytes to each other [7]. Despite the convergence in function, however, invertebrate immunoclotting-forming proteins and hemocytes hold little structural and phylogenetic similarities with hemostatic proteins and thrombocytes-platelets of vertebrate immunothrombosis [2,6]. Significantly, the first thrombin-catalyzed conversion of fibrinogen to fibrin coagulation system of vertebrates was assembled over a 50–100 million year window [2] in the earliest jawless marine vertebrates similar to today’s hagfish and lampreys (Figure 1), which possess prothrombin, tissue factor, fibrinogen, FVII, and X but lack genes for FVIII and FIX and the contact system [8,9]. These marine animals emerged at the onset of the Cambrian period [2,8] (Figure 1) roughly 540 million years ago (Mya). However, the Cambrian radiation, which might have been preceded by the first whole-genome duplication event [10,11], also witnessed the emergence of other physiological innovations, including a true endothelium, a closed and increasingly pressurized circulatory system, blood cell-type diversification, and the adaptive immune system (Figure 1) [11,12,13,14].

**Figure 2:**
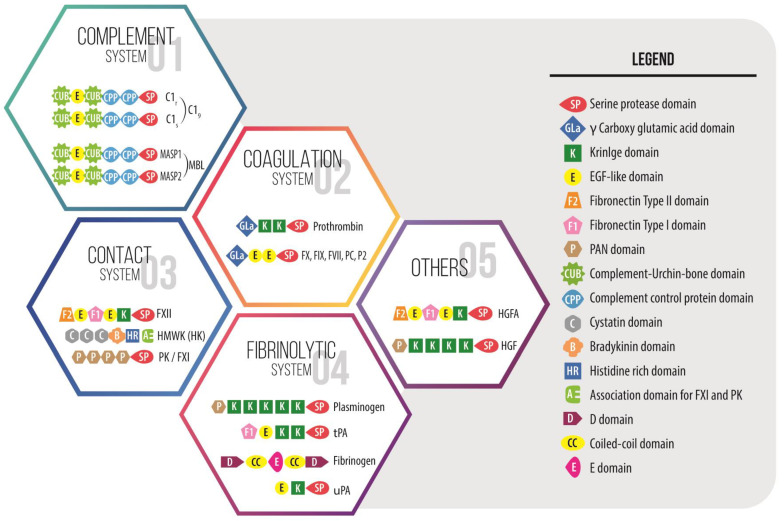
Domain organization of some multidomain proteins that make up the intravascular innate immune cascade systems with the catalytic serine protease domain as a key enzymatic region. These proteins share some domains with intrinsic chemical affinities useful in generating cooperative networks as pathogen and damage pattern recognition molecules, among other functions. Moreover, many of the proteins involved in coagulation and fibrinolysis are unique in that a large number of functions may coexist in one molecule and domain, which communicate signals across very different pathways. In these multifunctional molecules, the noncatalytic regions are involved in morphogen signaling, cell–cell and cell–matrix adhesion and interaction, thereby playing roles in other processes from embryonic development to inflammation to healing and beyond. The numbers in the figure are for illustrative purposes and do not correspond to the order of appearance from an evolutionary point of view.

This significant genomic event, together with gene duplication, exon shuffling, and simple mutation of preexisting genes, might have provided multicellular marine organisms with the genetic variations that led to the expansion of transcription factors and multidomain extracellular proteins, both key molecules for the radiation of multicellular organisms in the Cambrian period (Figure 2), including the materialization of the first version of the vertebrate coagulation system (Figure 1) [13,15,16].

## 3. The Emergence of Vertebrate Coagulation System and Hemostasis

The first vertebrate clotting components to arise were prothrombin (PT), followed by other Vitamin K-dependent clotting factor genes f10 and f7 in the earliest jawless marine vertebrates. The prothrombin precursor diverged from the preexisting complement and mannan-binding protein-associated serine proteases (C1r, C1s, and MASP-1, MASP-2, MASP-3) already present in deuterostomes, in which they were already operating as a primordial complement system (C3, Bf, and MASP) [17,18,19]. These trypsin-like serine proteases, in turn, were derived from the chymotrypsin-like serine protease domains with trypsin as the primordial gene [18,19], leading to amino acid replacement [2], similar to the origin of PT and other Vitamin K-dependent serine protease clotting factors (FVII, FIX, and FX) later on (Figure 2) [2,3,8]. The other essential substrate of vertebrate clots, fibrinogen, is not present in invertebrates, but fibrinogen domain-containing molecules are found in metazoa from sponges to mammals, with functions in development, agglutination, pathogen recognition, bacterial lysis, parasite defense, and allorecognition [20,21,22]. For instance, the invertebrate sea squirt possesses three genes that encode a protofibrinogen, which operates as a cell-based sealing protein by hemocyte agglutination as well as an immune enhancer with a prophylactic mechanism in trapping soluble parasite-derived molecules and in killing bacteria [2,21,23,24].

Since the Cambrian and during the Devonian period, and coincidentally with the start-up of the aforementioned physiological innovations (Figure 1) which led to the diversity of multi-systemic marine vertebrates, the simplest version of the coagulation system evolved towards the complex clotting machinery of mammals by conserving, adding and integrating other multidomain serine proteases in several stages [2]. This is the case in the human contact activation/kallikrein system (CAS) that initially evolved in marine vertebrates as an inflammatory kininogen–kinin formation system independently of fibrin forming events, to eventually come together with the appearance of tetrapods in the water-to-land transition (Figure 1) [2,8]. For instance, the high molecular-weight kininogen gene (*Kng1*) is present in all vertebrates, and the encoding protein, which encloses the bradykinin domain, expanded and acquired structural and functional complexity, from lampreys to humans, by domain acquisition and shuffling (Figure 2) [2,8,25]. As for the prekallikrein (PK) *klkb1* gene, which is present together with a simple version of *Kng1* in the coelacanth and lungfish, considerable evidence suggests that it is derived from plasminogen (PLG) serine protease and hepatocyte growth factor (HGF) genes (Figure 2), both already present in lampreys [2,8,25]. The central component of human CAS, the FXII gene (f12), emerged before the appearance of lungfishes through duplication of the chromosomal segment containing the pro-HGF activator (HGFAC) gene already present in cartilaginous fish (Figure 2) [8,25]. Finally, the merger of CAS with the fibrin formation pathway might have occurred with the appearance of tetrapods in the water-to-land transition 400–390 Mya (Figure 1) [2] since new research reveals that kallikrein protease (PKa) may directly activate factor IX with thrombin generation and fibrin formation independently of FXII and FXI [26]. This fact somehow challenges that the merger might have occurred late in vertebrate evolution, with the duplication of the klkb1 gene giving rise to the FXI gene (f11) in a monotreme or a proto-mammalian ancestor, as the FXI gene (f11) is present in platypi and opossums, but not in amphibians, reptiles, or birds (Figure 1) [8,25].

Another layer of complexity of the mammalian coagulation system was the emergence of the megakaryocyte/platelet axis (M/Pa). The comparison of hemostatic systems of egg-laying monotremes and marsupials does not show noticeable differences with eutherian M/Pa (Figure 1) [6], a fact that suggests that the emergence of the M/Pa might have occurred around 166 Mya. However, the series of genetic and ecological events giving rise to the M/Pa remains a puzzle, as is the polyploid feature of megakaryocytes [6]. It is likely that selective forces might have driven M/Pa selection due to the generation of resistant arterial plugs optimizing hemostasis in the high-pressure, high-flow conditions of the mammalian arterial system, the compartmentalization and avoidance of pathogen replication and dissemination through pathogen detection by toll-like receptor (TLR) 2 and 4, and the subsequent release of microbicidal thrombocidins and kinocidins [27,28] Appendix A. It has been suggested as well that M/Pa might have favored embryo implantation and avoided massive bleeding at the time of delivery, key events in mammal reproduction with invasive placenta [29].

## 4. Pleiotropic Effects of Proteins of the Coagulation System: From Coagulation and Inflammation to Healing

**Figure 3:**
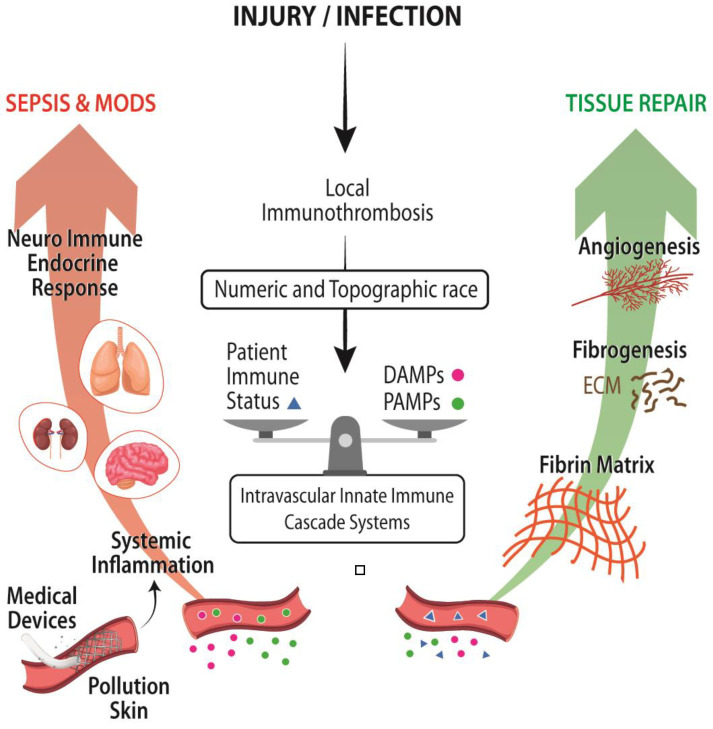
Immunothrombosis as a mosaic of defense mechanisms, including the activation of nociceptors, endothelial cells, fibroblasts, tissue-resident macrophages, circulating platelets and neutrophils, and plasma innate immune cascade systems, leads to curtailing and compartmentalizing the damage, thereby giving way to tissue repair. However, when the triggering emergency cannot be handled in a tissue autonomous manner (due to the patient status, a severe polytrauma, and/or the use of cutting-edge medical strategies), the numeric and topographic race between DAMPs and PAMPs, and the well-developed local checks tips the balance towards a gradual or massive entry of biotic and abiotic molecules into the bloodstream. In this new infectious or sterile inflammatory context, the blood-born cascade and the neuroendocrine systems may be systemically dysregulated, thereby causing a systemic inflammatory response syndrome (SIR). Moreover, the activation of the autonomic nervous system results in a barrier dysfunction that may lead to multiple organ dysfunction syndrome (MODS) and sepsis [33].

Injury, host defense responses, and healing appear to go hand in hand where intravascular proteolytic cascade systems play multiple roles in inflammation, coagulation, and healing. This is the case of thrombin that, besides its central role in blood coagulation, its signaling is necessary for limb, heart, and lens regeneration in the salamander and mammal’s local immunothrombosis. In wound healing, thrombin activates platelets, the IL-1α, and the HGFA-HGF-MET signaling pathways, the latter triggered as well by PK and FXII and functioning as a link between injury and repair [27,30,31]. Moreover, mammalian thrombin at low concentration deploys anti-inflammatory, antiapoptotic, and growth factor-like activities [27]. Similarly, vertebrate fibrin (ogen) protein executes multiple functions in addition to clotting formation from the immune modulation as an acute phase reactant to the defense against bacterial proliferation and dissemination by the generation of a fibrin biofilm in the air–liquid interface of the clot [32]. In addition, fibrin plays a crucial role in wound healing as a biodegradable provisional cell-friendly matrix with cell-attractant and cell-adhesion molecules trapped in the fibrin matrix and as a sink of morphogens and cytokines that bind to heparin sulfate binding domains and, once freed up, operate as cell-instructive molecules (Figure 3) [5].

FXII is another example of a multifunctional protein with pattern recognition and growth factor-like roles in inflammation, clotting, and wound healing [34]. Locally, the activation of FXII in wounds in the presence of soil particles abundant in silicates accelerates and strengthens fibrin-clotting formation and attracts and promotes neutrophil activities and NETosis, contributing to quickly compartmentalizing and sterilizing epithelial wounds [34,35]. This response prioritizes avoidance of the systemic spread of biotic elements in terrestrial high vertebrates (Figure 3) over the fast wound healing, with inflammatory neoangiogenic and profibrotic functions [34,35]. These FXII-mediated roles in skin epithelial wounds (and likely in the epithelium of lungs and gut) might have played a strong selection pressure, first in amphibians and then in reptiles and other terrestrial vertebrates, including mammals living in intertidal, muddy environments abundant in silicates [35]. This is not the case in some vertebrates with barely any contact with soil, such as cetaceans, which lost the klkb1 gene and possess an inactive f12 pseudogene, or birds, which lost the f12 gene [8]. Two other intravascular proteins, namely, HGFA and HGF, might have played immunothrombotic and repair roles in the water-to-land transition as they do in mammals. HGFA functions as a molecular link between tissue injury and repair through the HGFA-HGF-MET signaling pathway, whose activation is triggered by thrombin and CAS enzymes (PKa, FXII) [30]. The serine protease HGFA proteolytically activates HGF, resulting in pleiotropic HGF-mediated functions, from cell proliferation and differentiation to antifibrotic, angiogenic, anti-inflammatory, and regenerative activities [5,30]. Finally, activated platelets release biologically active proteins with significant roles in angiogenesis, fibrogenesis, cellular proliferation, migration and reprogramming, and resolution of inflammation, all of which are key processes in tissue repair and regeneration [27,36,37].

## 5. Vulnerabilities and Strengths of Immunothrombosis in Our Modern Medicine

The integration of immunothrombosis in mammalian organisms is represented by the interconnectedness of local short-range signaling pathways (complement, coagulation, contact systems, and fibrinolysis), complemented with and integrated into the systemic long-range neuroendocrine-immune signaling pathway, generated survival advantages [38]. However, this cooperative cross-talk among mosaic proteins such as TLR, Nod-like receptors (NLR), receptors for advanced glycation end products (RAGE), FXII, FVII, C1q, and Ficolins, among others, and present intracellularly in the fluid phase, and/or embedded in membranes of intravascular and tissue-resident cells, entails vulnerabilities [33]. This is the case when the local injury cannot be managed in a tissue autonomous manner (patient immune status, polytrauma and massive burns, hemorrhagic shock) (Figure 3). As a result, a gradual or massive amount of biotic and abiotic elements gain access to intravascular space, triggering the neuroendocrine stress response, which might result in a barrier dysfunction leading to an endotheliopathy, coagulopathy, immunoparesis, embedded into a systemic inflammatory response syndrome (SIRS) and multiple organ dysfunction syndrome (MODS) (Figure 3) [33].

These vulnerabilities apply as well to the exposure of blood to unscheduled and never anticipated medical devices and procedures (catheters, coronary stents, prosthetic heart valves, blood transfusions, extracorporeal circulation, and organ and tissue transplantations), a sedentary and prothrombotic lifestyle, and toxic environmental nanoparticles (from food supplements, cosmetics, vehicle exhaust and wearing of vehicle brakes, cigarettes, environmentally-caused wildfires) so unlike those conditions in which the emergence and integration of CAS evolved, fine-tuned, and operated (Figure 3) [39].

**Figure 4:**
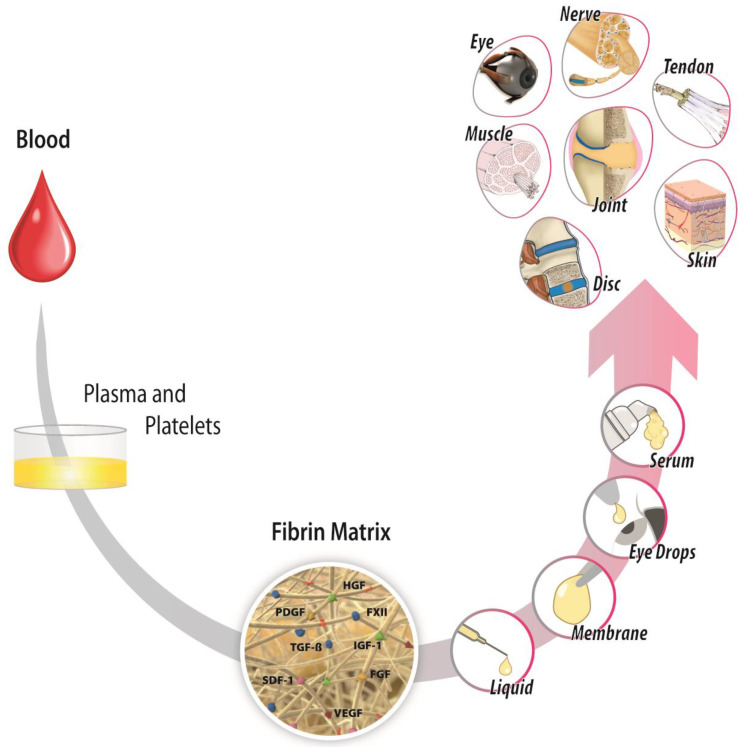
Autologous fibrin matrix (AFM) is a by-product of nature’s own healing systems that involves blood anticoagulation and mild centrifugation steps followed for fibrin formation by restoring thrombin production. The injected AFM into the damaged tissue operates as a liquid-to-gel dynamic scaffold that carries plasma- and platelet-derived growth factors, including TGF-β1, PDGF, VEGF, HGF, FXII, or IGF-1, as biological mediators for tissue repair. The gradual biodegradation of a fibrin clot mediated by the serine protease plasmin will release GFs that act as extracellular ligands by binding to transmembrane receptors on the surface of target cells, thereby activating intracellular signal transduction pathways to induce a wide range of cell specifications during inflammation and the healing process: cell survival, proliferation, migration, differentiation, transdifferentiation, maturation, and changes in protein synthesis and metabolism. These biologics are emerging as a promising therapeutic approach with antialgic, anti-inflammatory, trophic, antifibrotic, and tissue-specific angiogenesis effects. They are applied in sterile-inflammatory injuries such as osteoarthritis, tendinopathies, cartilage injuries, peripheral neuropathies, intervertebral disc degeneration, skin burns and ulcers, corneal ulcers, and dry eyes, among other conditions.

Blood has always been present in the equation of healing therapies. In fact, 80 years ago, Seddon and Medawar [40] implemented, in humans, a procedure called “fibrin suture” to carry out a median nerve repair, which led to sensory recovery outcome. Recently, other blood derivates, including autologous fibrin matrix (AFM), have emerged as by-products of nature’s own healing systems with local therapeutic uses in sterile inflammatory conditions (Figure 4) [5,27]. Basic science and clinical studies coming from the application of AFM or its supernatant indicate that AFM operates as a complex nonlinear dynamic network with antialgic, anti-inflammatory, trophic, antifibrotic, and tissue-specific angiogenesis effects [5,27]. AFM has been used in various medical specialties, from oral and maxillofacial surgery, to traumatology, orthopedic surgery and sports medicine, dermatology, ophthalmology, reproductive medicine, nerve regeneration, general surgery, and skin wound healing (Figure 4) [5,27].

## 6. Conclusions

It is highly likely that selection exerted strong pressure on the mechanisms underlying the early phases of immunothrombosis to operate quickly by linking immunity and coagulation as a key effective survival factor, leaving the immuno-reparative function as an open and condition-sensitive process aimed at functional recovery rather than at structural perfection. The paradox of host defense mechanisms acting as local tissue repair enhancers while in some cases generating systemic pathogenic processes remains insufficiently understood. However, the constitutive molecular pathways underlying immunothrombosis are a goldmine from which we can learn to design and optimize new bioengineering and synthetic biologically-based therapies. Therefore, blood is a source of biomolecules with pleiotropic effects, and AFM could be harnessed as a local therapeutic tool in sterile inflammatory conditions.

## Data Availability

Not applicable.

## Abbreviations

AFM	autologous fibrin matrix
CAS	contact activation/kallikrein system
HGF	hepatocyte growth factor
HGFAC	pro-HGF activator
M/Pa	megakaryocyte/platelet axis
MODS	multiple organ dysfunction syndrome
Mya	million years ago
NLR	Nod-like receptor
PK	prekallikrein
PKa	kallikrein protease
PLG	plasminogen
PT	prothrombin
RAGE	receptors for advanced glycation end products
SIRS	systemic inflammatory response syndrome
TLR	toll-like receptor

## Appendix A

This opinion article shares evidence already tackled in a previous article [27] published by our group. However, in this opinion article, we clearly focus our article on the evolutionary perspective of the coagulation system and healing, with emphasis on the domain composition of intravascular innate immune proteins. Both articles are complementary.
